# Variables affecting new drug prices in South Korea’s pricing system

**DOI:** 10.3389/fphar.2024.1370915

**Published:** 2024-05-09

**Authors:** Dong Yun Lee, Seong Ha Cho, Da Hye Lee, Su Jeong Kang, Jong Hyuk Lee

**Affiliations:** College of Pharmacy, Chung-Ang University, Seoul, Republic of Korea

**Keywords:** pricing, health technology assessment, drug price, budget impact, cost-effectiveness, patient accessibility

## Abstract

**Objective:** The price of pharmaceuticals is important from the economic and industrial perspectives but as well as patients’ access to treatment. This study aimed to analyze the variables affecting the prices of new drugs in South Korea’s pricing system.

**Methods:** Data on 192 new drugs listed in South Korea from 2012 to 2022 were collected from the official website of the Health Insurance Review and Assessment Service. The independent variables included drugs for severe diseases, alternatives, number of patients, number of advanced 7 countries listed, budget impact, and listing period. The dependent variables included annual treatment cost and the price ratio to the advanced 7 country’s average adjusted price. Descriptive statistics of variables, linear correlations between quantitative independent and dependent variables, and associations between independent and dependent variables were analyzed.

**Results:** The mean annual treatment cost and price ratio to the advanced 7 country’s average adjusted price were higher for drugs for severe diseases and those with no alternatives. Annual treatment cost and price ratio to the advanced 7 country’s average adjusted price were negatively correlated with the number of patients and positively correlated with the number of advanced 7 countries listed. Annual treatment cost was affected by the variables drugs for severe diseases, alternatives, number of patients, number of advanced 7 countries listed, and budget impact. The price ratio to the advanced 7 country’s average adjusted price was affected by drugs for severe diseases, alternatives, and the number of patients.

**Conclusion:** This study revealed the effect of different variables on the prices of new drugs in South Korea, allowing for the development of a more effective assessment system to evaluate the prices of new drugs while ensuring profitability for pharmaceutical companies, sustainability of public insurance, and accessibility to drugs by patients.

## 1 Introduction

In the healthcare system, the price of pharmaceuticals is important not only from the economic and industrial perspectives but also in terms of patients’ access to treatment (Lotvin et al., 2014; Phelan and Cook, 2014; Vogler et al., 2015). Specifically, the price of new drugs directly affects pharmaceutical companies’ profit generation and motivation for new drug development and considerably impacts patients’ access to novel treatments (Vincent Rajkumar, 2020). Moreover, from a payer’s perspective, if the price of a new drug is higher than its intrinsic value, efficient resource allocation may not occur. This could threaten the sustainability of the health insurance owing to budgetary constraints, and therefore, it is crucial to carefully determine the price of new drugs (National Academies of Sciences et al., 2019). Especially in countries that offer public health insurance, determining the reimbursement price for new drugs considerably affects insurance finances and patient accessibility. Consequently, conflicts among stakeholders (including patients, medical professionals, pharmaceutical companies, and payers) on the pricing issue are common. Therefore, establishing a fair and efficient pricing system through social consensus among all stakeholders is crucial.

Although different countries have different methods for setting the price of new drugs, they generally consider factors such as clinical usefulness, cost-effectiveness, disease prevalence, disease characteristics, societal demand, and potential economic effects (Onakpoya et al., 2015; Vogler et al., 2017; Tafuri et al., 2022).

South Korea provides public health insurance to each individual. For a new drug to be reimbursed under the health insurance system, it must prove its cost-effectiveness (MOHW, 2006; Park et al., 2012). Health technology assessment (HTA) is employed to evaluate the cost-effectiveness of new drugs or medical technologies, supporting the efficient use of medical resources. However, assessing innovative, high-priced drugs that are being developed using the traditional HTA method may be challenging, preventing them from being covered under public insurance. Hence, considering the parameters other than cost-effectiveness that reflect various societal factors, such as unmet needs, patient population characteristics, social demand, ethical considerations, innovation and value, economic effects, and the impact on the industry is necessary to ensure patient access to new drugs (Janssen Daalen et al., 2021; Żelewski et al., 2022). Accordingly, South Korea has introduced measures to mitigate the limitations of the traditional HTA. These measures include exemption from the evaluation of cost-effectiveness for certain drugs, flexible application of incremental cost-effectiveness ratio thresholds, and risk sharing agreements (RSAs) to increase access to advanced treatments (Lee, 2021).

**FIGURE 1:**
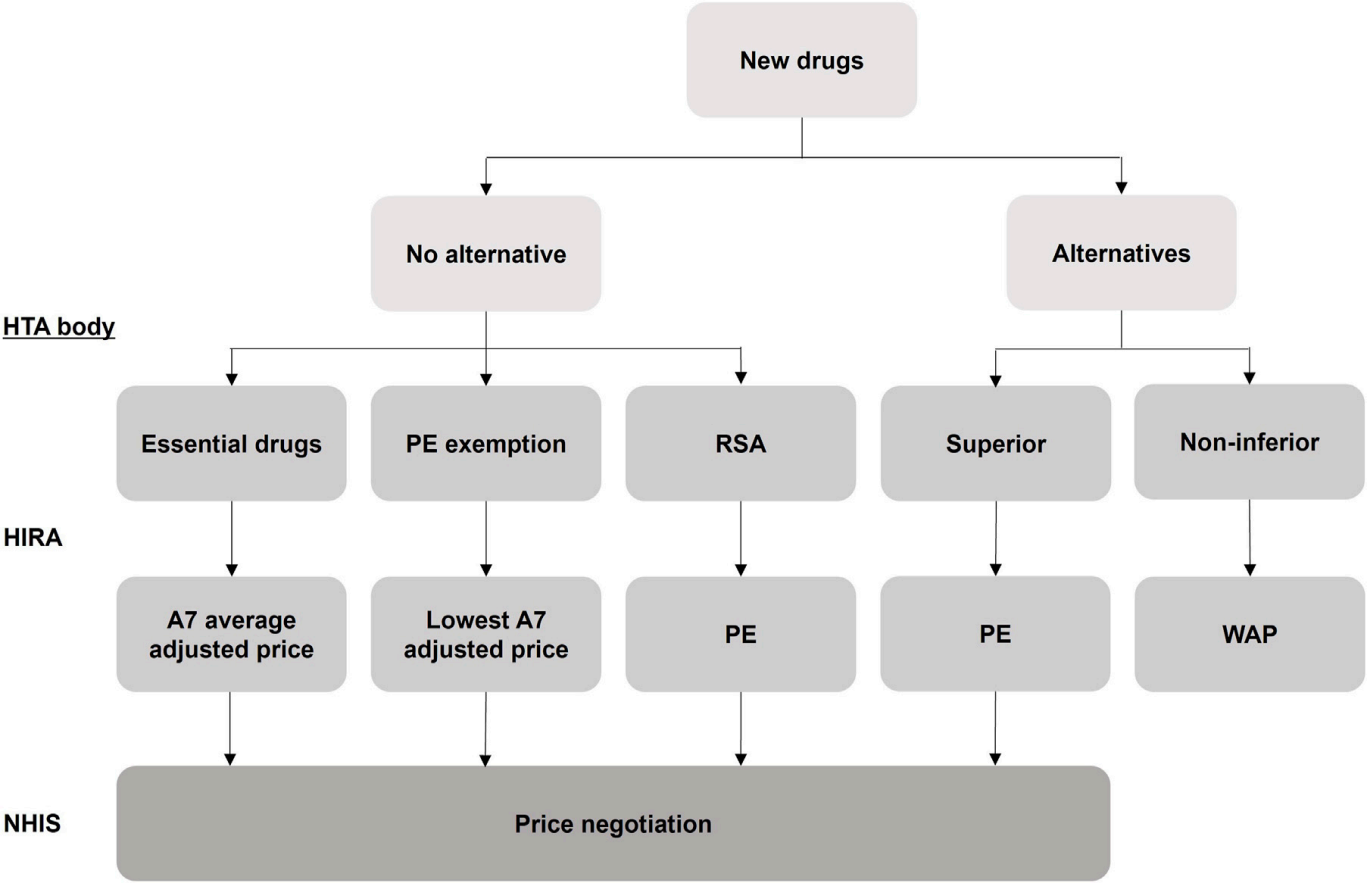
Evaluation scheme for the pricing of new drugs in South Korea. HTA: Health Technology Assessment; HIRA: Health Insurance Review and Assessment Service; PE: Pharmacoeconomic Evaluation; RSA: Risk Sharing Agreement; WAP: Weighted Average Price; NHIS: National Health Insurance Service. A7 (countries): 7 advanced countries (the United States, the United Kingdom, Germany, France, Italy, Switzerland, and Japan).

South Korea’s pricing system is divided into two pathways depending on the availability of alternatives (Kim et al., 2021). If there are alternatives, clinical usefulness is evaluated and if superior to the alternatives, cost-effectiveness is reviewed through pharmacoeconomic evaluation (PE). If the clinical usefulness is evaluated and is non-inferior to the alternatives, the drug price is determined by the weighted average price of the alternatives without price negotiation. If there are no alternatives, three pricing pathways can be considered: essential drugs, PE exemption, and RSA. In the case of a RSA, cost-effectiveness is reviewed through PE. The drug price is determined through price negotiation by referring to the advanced 7 country’s (A7: the United States, the United Kingdom, Germany, France, Italy, Switzerland, and Japan) average adjusted price for essential drugs, the lowest A7 adjusted price for PE exemption, and the premium price compared to alternatives for PE (Figure 1).

A majority of the studies on the factors affecting the pricing of new drugs focused on medications used for specific disease groups, such as orphan and oncology drugs. In Italy, studies have analyzed the annual treatment cost or budget impact of orphan and oncology drugs (Jommi et al., 2021; Russo et al., 2021; Villa et al., 2022; Manea et al., 2023). Additionally, correlations between the treatment costs of orphan drugs and the prevalence and incidence of the respective diseases were also examined. Worm and Dintsios found statistically significant associations between the price of orphan drugs and i) the therapeutic area, ii) approval for pediatric care, iii) treatment population size, iv) cost of comparative therapies, and v) European prices, with European prices showing the strongest correlation with orphan drug prices (Worm and Dintsios, 2020). Furthermore, various studies on topics related to drug pricing, including analyses of determinants affecting drug prices and comparisons of ex-factory prices per unit, have been conducted in Japan, Australia, and the United Kingdom (Jørgensen and Kefalas, 2016; Vogler et al., 2016; Young et al., 2017; Trotta et al., 2019; Gandjour et al., 2020; Mamiya and Igarashi, 2021; Kawakami and Masamune, 2022). One study compared the prices of oncology drugs in South Korea, listed from 2007 to 2017, with those in A7 countries, to assess patient accessibility to novel oncology drugs (Jung et al., 2021). Although numerous studies have explored the variables affecting the price of new drugs, empirical research on what factors influence the decision-making involved in setting new drug prices in South Korea has not yet been conducted.

This study investigated the factors that influence the pricing of new drugs in the South Korean health insurance system. Unlike previous studies that focused on specific categories of drugs, such as oncology or orphan drugs, this study empirically examined all new drugs reimbursed in South Korea over the last decade to determine the factors influencing their pricing.

## 2 Materials and methods

### 2.1 Study design

Our data set covers 192 new drugs listed in South Korea during the period of 2012–2022. We split the methods into the parts of descriptive and statistical analysis. In consideration of the South Korean pricing and reimbursement system, 6 independent variables were selected, and annual treatment cost and price ratio to the A7 average adjusted price of new drugs were used as dependent variables representing prices. Student’s t-test, Pearson’s correlation coefficients, and multiple linear regression analysis were used to test the effects of these independent variables on the price of new drugs.

### 2.2 Data collection

Data on 192 new drugs listed in South Korea from 2012 to 2022 were extracted from the official website of the Health Insurance Review and Assessment Service (HIRA, 2023c). The information on the variables included in this study was obtained from the Drug Reimbursement and Evaluation Committee reports by the Health Insurance Review and Assessment Service (HIRA, 2023a). New drugs for which information on one or more variables was not disclosed in the reports were excluded from the study.

#### 2.2.1 Independent variables

Factors that are mainly considered when determining the price of new drugs in terms of clinical usefulness, cost-effectiveness, budget impact, and external reference pricing were selected as the following independent variables.

Drugs for severe diseases: drugs for rare diseases or cancer.

Alternatives: drugs of an equivalent therapeutic level.

Number of patients: number of patients with the target disease.

Number of A7 countries listed: number of countries listed among the 7 advanced countries.

Budget impact: expected annual claim amount against the new drugs.

Listing period: period between the marketing authorization date and the listing date.

#### 2.2.2 Dependent variables

##### 2.2.2.1 Annual treatment cost

The annual treatment cost was calculated based on the listed price, dosage and treatment schedule recommended by the Ministry of Food and Drug Safety, and 1 year of treatment duration unless a shorter time was envisaged (for example, for one-shot therapies). If the drug had more than one indication, the first one, in order of approval time, was considered.

##### 2.2.2.2 Price ratio to the A7 average adjusted price

The price ratio to the A7 average adjusted price was calculated by dividing the listed price in South Korea by the average adjusted price of the drug in the A7 countries. When South Korea reference other countries’ price, price is adjusted considering the difference in price structure between South Korea and other countries. The adjusted price is calculated by applying the ex-factory rate to the price identified on drug price websites of the A7 countries [the United States’s Red book, the United Kingdom’s Monthly Index of Medical Specialities, Germany’s Rote Liste, France’s Vidal, Italy’s L'Informatore Farmaceutico, Switzerland’s Arzneimittel Kompendium, and Japan’s Hokenyaku Jiten (Yakugyo Kenkyukai)], and then applying the exchange rate, value-added tax and distribution margin (HIRA, 2023b). New drugs developed in South Korea were not considered.

### 2.3 Data analysis

Characteristics of the new drugs considered in this study were analyzed using descriptive statistics. Quantitative dependent variables, based on categorical independent variables, were analyzed using the Student’s t-test. Linear correlations between quantitative independent and dependent variables were analyzed using Pearson’s correlation coefficients. Associations between independent variables and quantitative dependent variables were analyzed using multiple linear regression analysis. The number of patients per 100,000, log-transformed budget impact, listing period in years, and log-transformed annual treatment cost were considered in the study. Log-transformation was used to deal with positively skewed data. All analyses were performed using SPSS version 29.0 (SPSS Inc., Chicago, United States). All *p*-values were two-tailed, and *p* < 0.05 was considered statistically significant.

## 3 Results

**FIGURE 2:**
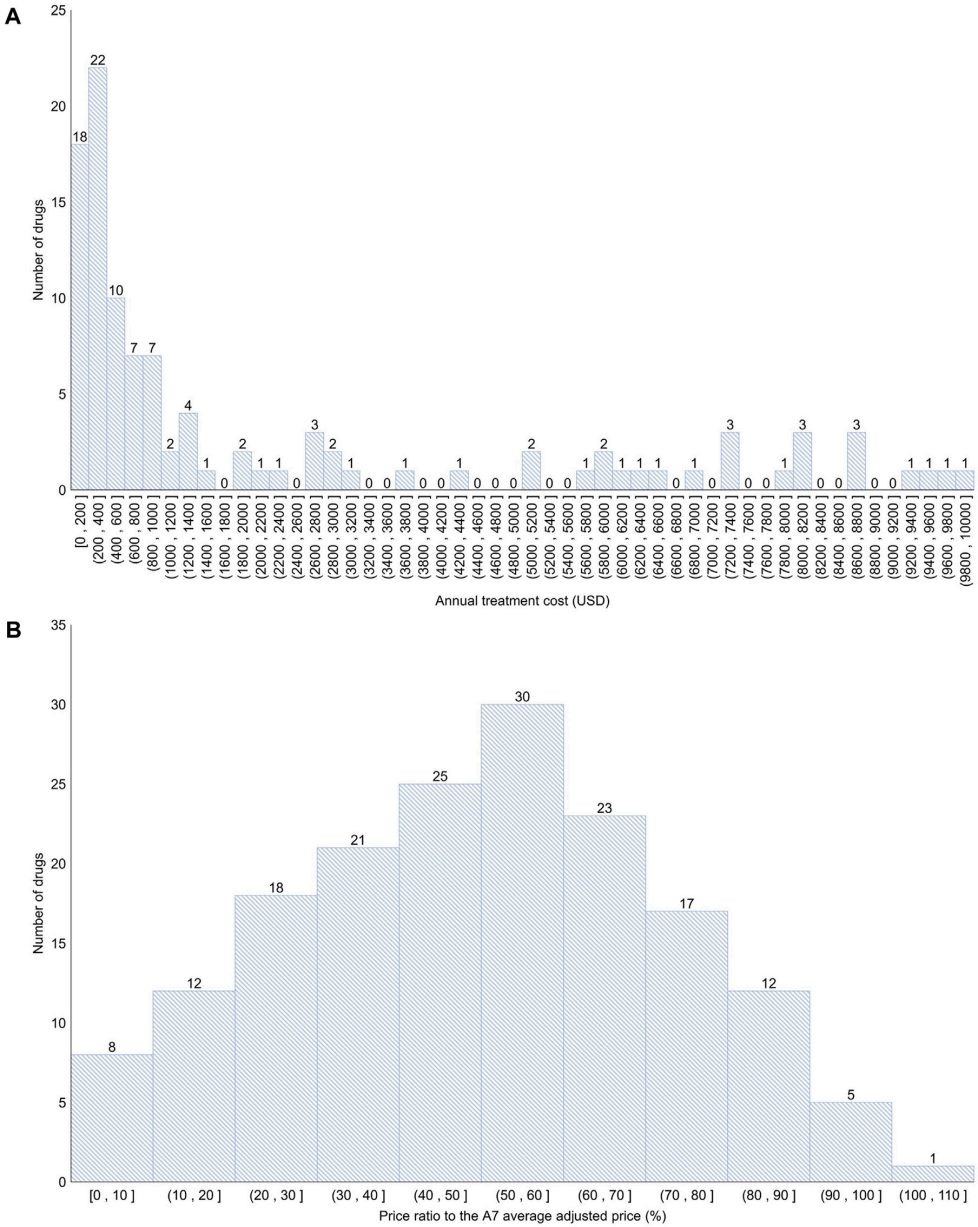
Distribution of annual treatment cost and the price ratio to the A7 average adjusted price **(A)** < 10,000 USD (106 products; 55.2%); >10,000 USD (86 products; 44.8%). **(B)** Approximately 0%–50% (84 products; 43.8%); approximately 50%–100% (87 products; 45.3%); >100% (1 product; 0.5%). A7 (countries): 7 advanced countries (the United States, the United Kingdom, Germany, France, Italy, Switzerland, and Japan).

Among the 192 new drugs included in this study, 90 (46.9%) were for severe diseases and 43 (22.4%) did not have any alternatives. For these 192 drugs, the mean number of patients was 281,693 [standard deviation (SD) = 980,943], number of A7 countries listed was 4 (SD = 2), budget impact was 11,710,000 USD (SD = 15,232,000 USD), listing period was 717 days (SD = 777 days), annual treatment cost was 36,047 USD (SD = 82,678 USD), and the price ratio to the A7 average adjusted price was 49.6% (SD = 23.3%) (Table 1; Figure 2).

**TABLE 1:** Descriptive statistics of variables related to the price of new drugs in South Korea.

	Categorical variables	No		Yes	
Independent variables (N = 192)	Drugs for severe diseases	102 (53.1%)		90 (46.9%)	
	Alternatives	43 (22.4%)		149 (77.6%)	
	Quantitative variables	Mean	SD	Median	Min–Max
	Number of patients	281,693	980,943	2,907	14–8,163,770
	Number of A7 countries listed	4	2	5	0–7
	Budget impact	11,710	15,232	6,105	71–96,184
	Listing period	717	777	432	0–4,529
Dependent variables (N = 192)	Annual treatment cost	36,047	82,678	7,998	1–629,432
	Price ratio to the A7 average adjusted price	49.6	23.3	51.0	2.6–105.7

The units of budget impact, listing period, and annual treatment cost are thousand USD, d, and USD, respectively. USD/KRW, exchange rate is 0.00075 (1 September 2023). A7 (countries): 7 advanced countries (the United States, the United Kingdom, Germany, France, Italy, Switzerland, and Japan).

**FIGURE 3:**
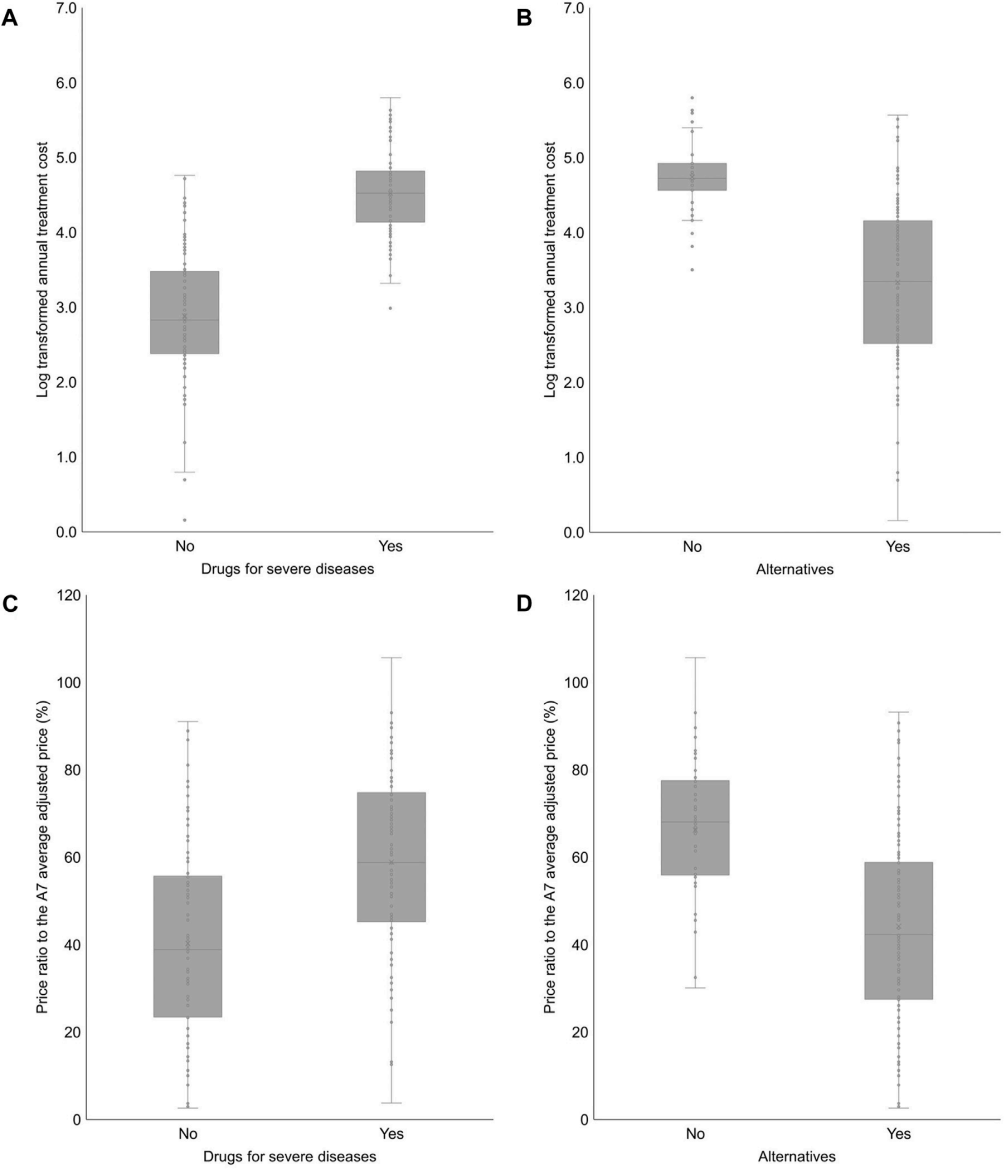
Annual treatment cost and the price ratio to the A7 average adjusted price according to categorical independent variables **(A)** 2.882 ± 0.884 (0.156–4.761) vs. 4.522 ± 0.543 (2.985–5.799); *p* < 0.001. **(B)** 4.739 ± 0.475 (3.502–5.799) vs. 3.337 ± 1.036 (0.156–5.566); *p* < 0.001. **(C)** 40.3 ± 22.0 (2.6–91.0) vs. 58.9 ± 20.8 (3.8–105.7); *p* < 0.001. **(D)** 66.2 ± 16.2 (30.1–105.7) vs. 44.2 ± 22.7 (2.6–93.2); *p* < 0.001. A7 (countries): 7 advanced countries (the United States, the United Kingdom, Germany, France, Italy, Switzerland, and Japan).

The annual treatment cost of drugs for severe diseases was significantly higher than that of drugs not for severe diseases (2.882 vs. 4.522; log-transformed; *p* < 0.001). Additionally, the annual treatment cost of drugs with no alternatives was significantly higher than that of drugs with alternatives (4.739 vs. 3.337; log-transformed; *p* < 0.001). The price ratio to the A7 average adjusted price of drugs for severe diseases was significantly higher than that of drugs not for severe diseases (40.3% vs. 58.9%; *p* < 0.001). The price ratio to the A7 average adjusted price of drugs with no alternatives was significantly higher than that of drugs with alternatives (66.2% vs. 44.2%; *p* < 0.001) (Figure 3).

The annual treatment cost was negatively correlated with the number of patients (Pearson’s correlation coefficient = −0.411; *p* < 0.001) but positively correlated with the number of A7 countries listed (Pearson’s correlation coefficient = 0.404; *p* < 0.001) and the listing period (Pearson’s correlation coefficient = 0.172; *p* = 0.018). The price ratio to the A7 average adjusted price was negatively correlated with the number of patients (Pearson’s correlation coefficient = −0.341; *p* < 0.001) and positively correlated with the number of A7 countries listed (Pearson’s correlation coefficient = 0.173; *p* = 0.023) (Table 2).

**TABLE 2:** Correlation between annual treatment cost or the price ratio to the A7 average adjusted price and quantitative independent variables.

Quantitative independent variables	Annual treatment cost	Price ratio to the A7 average adjusted price
Number of patients	−0.411 (*p* < 0.001)	−0.341 (*p* < 0.001)
Number of A7 countries listed	0.404 (*p* < 0.001)	0.173 (*p* = 0.023)
Budget impact	0.231 (*p* = 0.009)	0.033 (*p* = 0.671)
Listing period	0.172 (*p* = 0.018)	0.146 (*p* = 0.057)

The results of correlation analysis are represented by Pearson’s correlation coefficients. A7 (countries): 7 advanced countries (the United States, the United Kingdom, Germany, France, Italy, Switzerland, and Japan).

The annual treatment cost was affected by the following variables: drugs for severe diseases, alternatives, number of patients, number of A7 countries listed, and budget impact (*R* ^2^ = 0.672; Durbin-Watson = 1.922; *p* < 0.001). The price ratio to the A7 average adjusted price was affected by the variables drugs for severe diseases, alternatives, and number of patients (*R* ^2^ = 0.262; Durbin-Watson = 2.087; *p* < 0.001) It was confirmed that the correlation between independent variables was not significant based on the variance inflation factor of 1–10 (Table 3).

**TABLE 3:** Association between annual treatment cost or the price ratio to the A7 average adjusted price and independent variables.

Dependent variables	Independent variables	Coefficient	*p*-value	VIF
Annual treatment cost	Drugs for severe diseases	1.223	*p* < 0.001	1.580
	Alternatives	−0.359	*p* = 0.007	1.456
	Number of patients	−0.022	*p* < 0.001	1.195
	Number of A7 countries listed	0.064	*p* = 0.005	1.284
	Budget impact	0.396	*p* < 0.001	1.034
Price ratio to the A7 average adjusted price	Drugs for severe diseases	8.116	*p* = 0.032	1.494
	Alternatives	−14.274	*p* < 0.001	1.409
	Number of patients	−0.946	*p* < 0.001	1.089

VIF: Variance Inflation Factor. A7 (countries): 7 advanced countries (the United States, the United Kingdom, Germany, France, Italy, Switzerland, and Japan).

## 4 Discussion

Setting an appropriate price for new drugs is crucial not only to guarantee profits for pharmaceutical companies but also to ensure their continued investment in research and development. This, in turn, influences the accessibility to these new drugs for patients. If the price of a new drug is overvalued, it can strain the fiscal health and lead to increased out-of-pocket expenses for patients, thereby reducing their access to treatments in countries providing a public health insurance system (Parker-Lue et al., 2015). Therefore, a system where new drugs are swiftly and appropriately reimbursed is indispensable within the public healthcare system. In South Korea, the price of new drugs is determined by adjusting the average price of the drug in the A7 countries, resulting in relatively low listing prices. An analysis of the prices of 192 new drugs listed in South Korea from 2012 to 2022 showed that the median and average annual treatment cost was approximately 8,000 USD and 36,000 USD, respectively, whereas, the mean of the price ratio to the A7 average adjusted price ranged from 3% to 50% for 84 drugs (43.8%), 50%–100% for 87 drugs (45.3%), and 106% for 1 drug (0.5%). Whether the current pricing level in South Korea can simultaneously ensure the promotion of research and development by the pharmaceutical companies, fiscal health, and patient accessibility is unclear and requires further monitoring.

Korchagina et al. reported that the availability of alternatives impacts the price of orphan drugs, and drugs without alternatives are priced higher than those with alternatives (Korchagina et al., 2017). Treatments for rare diseases are concentrated around metabolic and hematological diseases, raising concerns about other rare diseases that are therapeutically neglected. Drugs for diseases lacking adequate treatment options with a high societal demand for the same tend to be overpriced (Manea et al., 2023). Our study also revealed that drugs for severe diseases and those without alternatives had higher annual treatment costs and a higher price ratio to the A7 average adjusted price. This suggests that societal demand and unmet needs are considered during drug pricing decisions in South Korea.

Gandjour et al. reported that drugs used for diseases with a small target population size tended to have a higher annual treatment cost (Gandjour et al., 2020). According to Jørgensen and Kefalas, the price discrepancy for high-cost therapies targeting small patient populations was less (Jørgensen and Kefalas, 2016). Onakpoya et al. indicated that drugs used for diseases with a low prevalence tended to have higher annual costs (Onakpoya et al., 2015). Similarly, Korchagina et al. reported that drugs used for diseases with a low prevalence tended to have a higher annual treatment cost than those with a high prevalence (Korchagina et al., 2017). Jommi et al. found that the lower the prevalence of a disease, the higher the respective annual treatment cost. Moreover, for diseases with a prevalence below the median, the presence of randomized controlled trials was associated with a higher annual treatment cost (Jommi et al., 2021). Considering the heterogeneity and small size of the patient population and the uncertainty that remains even after conducting randomized controlled trials, some European countries offer more flexibility in the benefit assessment of orphan drugs, exempting the use of standard HTA for orphan drugs, with the budget impact considered over cost-effectiveness in certain cases (Tafuri et al., 2022). Likewise, in this study, we observed that the lesser the number of patients, the higher was the annual treatment cost and price ratio to the A7 average adjusted price. This suggests that similar to other countries, disease prevalence is also considered while determining the price of new drugs in South Korea.

The results of this study indicated that if more countries listed a particular drug, the annual treatment cost and price ratio to the A7 average adjusted price tended to increase. South Korea, which enforces external reference pricing, substantially considers foreign reimbursement evaluations (Kim et al., 2021). Therefore, drugs listed in numerous A7 countries are likely priced higher in South Korea. Many global big-pharma products are listed in multiple A7 countries. In Europe, after receiving EMA approval, drugs undergo a national reimbursement evaluation process. Therefore, when such drugs are listed in South Korea, they have likely already been evaluated for reimbursement in other major countries, leading to potentially higher prices owing to greater room for negotiation and high evidence of efficacy.

According to the study by Korchagina et al., the shorter the delay between the HTA and drug commercialization, the higher the annual treatment cost. If there are complex negotiations and disagreements regarding the product value, the time between the HTA and drug commercialization can be extended (Korchagina et al., 2017). This means that for drugs used for severe diseases or those with high unmet needs, the government may accept a higher price for faster access due to social demands, such as necessity in clinical settings. According to the Patients Waiting to Access Innovative Therapies Indicator 2020 Survey conducted by the European Federation of Pharmaceutical Industries and Associations, orphan drugs have an EU average availability that is 8% lower, and the average time for availability is 5 months longer. This can be attributed to the fact that many orphan drugs are often subjected to a managed entry agreement, leading to significant time consumption (Tafuri et al., 2022).

Regarding the listing of new drugs in South Korea, it was observed that the longer the listing period, the higher the annual treatment cost. This can be attributed to the fact that drugs with higher prices tend to have longer processes for RSA, cost-effectiveness evaluation, and price negotiations, and conflicting positions among stakeholders often exist. Innovative drugs targeting severe diseases with high unmet needs are more likely to have higher prices. It is essential to establish a pricing system that allows drugs with high social demand to be listed swiftly.

This study had several limitations. Drugs for which an RSA had been concluded could not reflect the difference between the listed and actual prices owing to the low accessibility to information on actual prices. Furthermore, the calculation of the annual medication cost was based on the approval from the Korea Ministry of Food and Drug Safety. However, in clinical settings, prescriptions are based on clinical practice guidelines and reimbursement criteria. As a result, the inability to standardize this could lead to a variation from the actual data. PEs, RSAs, and the flexible incremental cost-effectiveness ratio threshold system, which can influence prices in South Korea’s pricing system, were not included in the scope of this study either. Further research on these aspects is warranted. A comparison of the drug pricing systems of South Korea and other countries should also be conducted.

Intensive social discussions among stakeholders are necessary to ensure that the inherent value of the drug is accurately reflected, and that the pricing system is operated in a way that enhances access to treatment. Particularly for drugs with novel modalities, such as one-shot cell and gene therapy, traditional drug pricing strategies based on cost-effectiveness are limited in their assessment of the drug’s value. Thus, the establishment of appropriate evaluation methods for these drugs is needed. Essentially, with the innovation of pharmaceuticals, there is a pressing need to appropriately incorporate social values such as economic benefits and the impact on the industry, ensuring the sustainability of finances. Therefore, the establishment of an innovative pricing system for these new drugs is essential. This study can provide evidence for stakeholders involved in pricing decisions, serve as a reference for pharmaceutical companies interested in introducing their drugs in South Korea, and be a valuable resource for researchers studying the South Korean pricing system and stakeholders in countries with similar pricing systems.

## Data Availability

The datasets analyzed in this study are available from the corresponding author upon reasonable request.
