# Supplementary material for: Variables affecting new drug prices in South Korea’s pricing system
Source: Front Pharmacol. 2024 May 9;15:1370915. doi: 10.3389/fphar.2024.1370915 (PMC11113548; doi:10.3389/fphar.2024.1370915)
Supplement: Supplementary file 1 [file DataSheet1.DOCX]

Supplementary Material


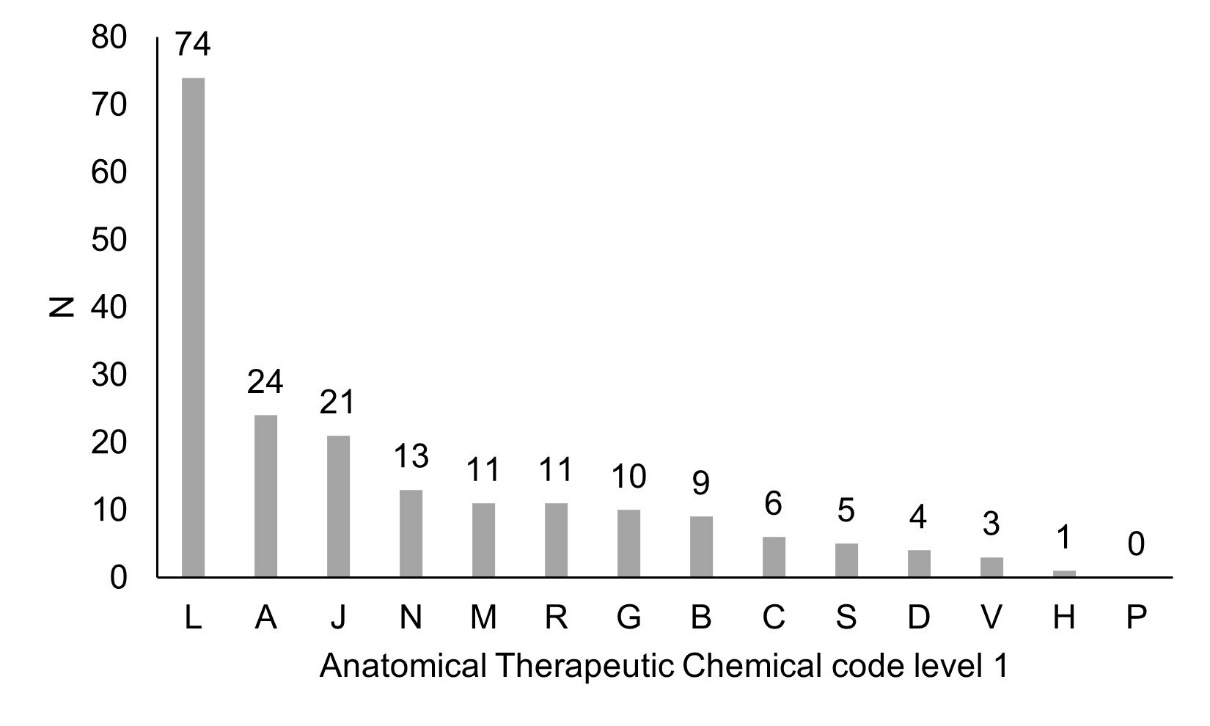


**Supplementary Figure 1.** Number of new drugs considered in this study according to Anatomical Therapeutic Chemical code level 1

A: alimentary tract and metabolism; B: blood and blood forming organs; C: cardiovascular system; D: dermatologicals; G: genito urinary system and sex hormones; H: systemic hormonal preparations, excl. sex hormones and insulins; J: antiinfectives for systemic use; L: antineoplastic and immunomodulating agents; M: musculo-skeletal system; N: nervous system; P: antiparasitic products, insecticides and repellents; R: respiratory system; S: sensory organs; V: various.
